# Fluorescence-Guided Surgery: A Review on Timing and Use in Brain Tumor Surgery

**DOI:** 10.3389/fneur.2021.682151

**Published:** 2021-06-16

**Authors:** Alexander J. Schupper, Manasa Rao, Nicki Mohammadi, Rebecca Baron, John Y. K. Lee, Francesco Acerbi, Constantinos G. Hadjipanayis

**Affiliations:** ^1^Department of Neurosurgery, Icahn School of Medicine at Mount Sinai, New York, NY, United States; ^2^Department of Neurosurgery, University of Pennsylvania School of Medicine, Philadelphia, PA, United States; ^3^Department of Neurosurgery, Fondazione Istituto Di Ricovero e Cura a Carattere Scientifico Istituto Neurologico Carlo Besta, Milan, Italy

**Keywords:** fluorescence-guided surgery, 5-ALA, fluorescein, ICG, extent of resection, timing

## Abstract

Fluorescence-guided surgery (FGS) allows surgeons to have improved visualization of tumor tissue in the operating room, enabling maximal safe resection of malignant brain tumors. Over the past two decades, multiple fluorescent agents have been studied for FGS, including 5-aminolevulinic acid (5-ALA), fluorescein sodium, and indocyanine green (ICG). Both non-targeted and targeted fluorescent agents are currently being used in clinical practice, as well as under investigation, for glioma visualization and resection. While the efficacy of intraoperative fluorescence in studied fluorophores has been well established in the literature, the effect of timing on fluorophore administration in glioma surgery has not been as well depicted. In the past year, recent studies of 5-ALA use have shown that intraoperative fluorescence may persist beyond the previously studied window used in prior multicenter trials. Additionally, the use of fluorophores for different brain tumor types is discussed in detail, including a discussion of choosing the right fluorophore based on tumor etiology. In the following review, the authors will describe the temporal nature of the various fluorophores used in glioma surgery, what remains uncertain in FGS, and provide a guide for using fluorescence as a surgical adjunct in brain tumor surgery.

## Introduction

The single best prognostic factor for patients diagnosed with high-grade gliomas (HGGs) is maximal resection, also known as gross total surgical resection (GTR) (1–3). HGG GTR has been associated with greater overall survival in addition to longer progression-free survival (PFS) (1, 4).In order to maximize the extent of resection (EOR), a number of visualization techniques have been introduced into the field of neurosurgery. Fluorescent agents, also known as fluorophores, aid in the delineation of normal and malignant tumor tissue (5, 6), permitting real-time image guided surgery that can maximize EOR (7).

There are a limited number of fluorophores currently used in clinical practice. Stummer first described the use of 5-aminolevulinic acid (5-ALA) and FGS in glioma patients in 1998. Based on the results of a landmark randomized, Phase III study confirming greater tumor resection and better patient outcomes in comparison to conventional microsurgery, 5-ALA (Gleolan) was approved as an oral intraoperative imaging agent for visualization of malignant tissue during glioma surgery (8–10). The fluorophores have various mechanisms of action, ranging from intracellular uptake with 5-ALA (11, 12) and ICG (13) to extracellular accumulation with fluorescein sodium (FS) and indocyanine-green (ICG), similar to the contrast enhancement found with gadolinium contrast enhancement found with MR imaging (14–16). FS is administered systemically after the induction of anesthesia while ICG and 5-ALA are given several hours before surgery (11, 15, 17, 18) (Table 1).

**Table 1:** Summary of the properties of fluorophores currently used in fluorescence-guided surgery.

**Agent**	**Excitation (nm)**	**Emission (nm)**	**Targeting mode**	**Administration mode**	**Dosage (in humans)**	**Half-life**	**Time prior to visualization**	**Time to fluorescence disappearing in target tissue**
ICG	778	700–850	Passive	IV	0.2–5 mg/kg	3–4 min	Seconds	Several minutes
Second Window ICG	778	700–850	Passive	IV	2.5–5.0 mg/kg		24 h	>72 h
Fluorescein	460–500	540–690	Passive	IV	2–20 mg/kg	23.5 min	2–4 h	2–4 h
5-ALA	375–440	640–710	Metabolic	Oral	20 mg/kg	1–3 h	2–8 h	22 h
BLZ-100	785	700–850	Molecular	IV	3–30 mg	30 min	3–29 h	48 h
CLR1501	500	517	Molecular	IV	16 mg/kg	4 days	4 days	–
CLR1502	760	778	Molecular	IV	2 mg/kg	4 days	4 days	–
IRDye800CW (EGFR)	–	794	Molecular	IV	Up to 24.5 mg/kg	15–20 min	1 h	3–4 days

*ICG, indocyanine green; 5-ALA, 5-aminolevulinic acid; IV, intravenous*.

While each of these agents used for FGS are currently used in patients, there is still ongoing research on the pharmacokinetics of these molecules in order to understand the optimal timing of administration and fluorescence of tumors during surgery. Timing is important with fluorophore administration, as it can be impacted by patient and surgical delays, and may affect the efficacy of the agent. Other new targeted fluorophores are currently being investigated in HGG patients and also require better understanding of optimal administration for FGS. The purpose of this review is to elucidate the current evidence on the perspective of timing for the various fluorophores used in glioma patients for FGS and provide an outline of the literature on the use of the most currently used fluorophores for glioma surgery.

## Indocyanine Green (ICG)

**Figure 1:**
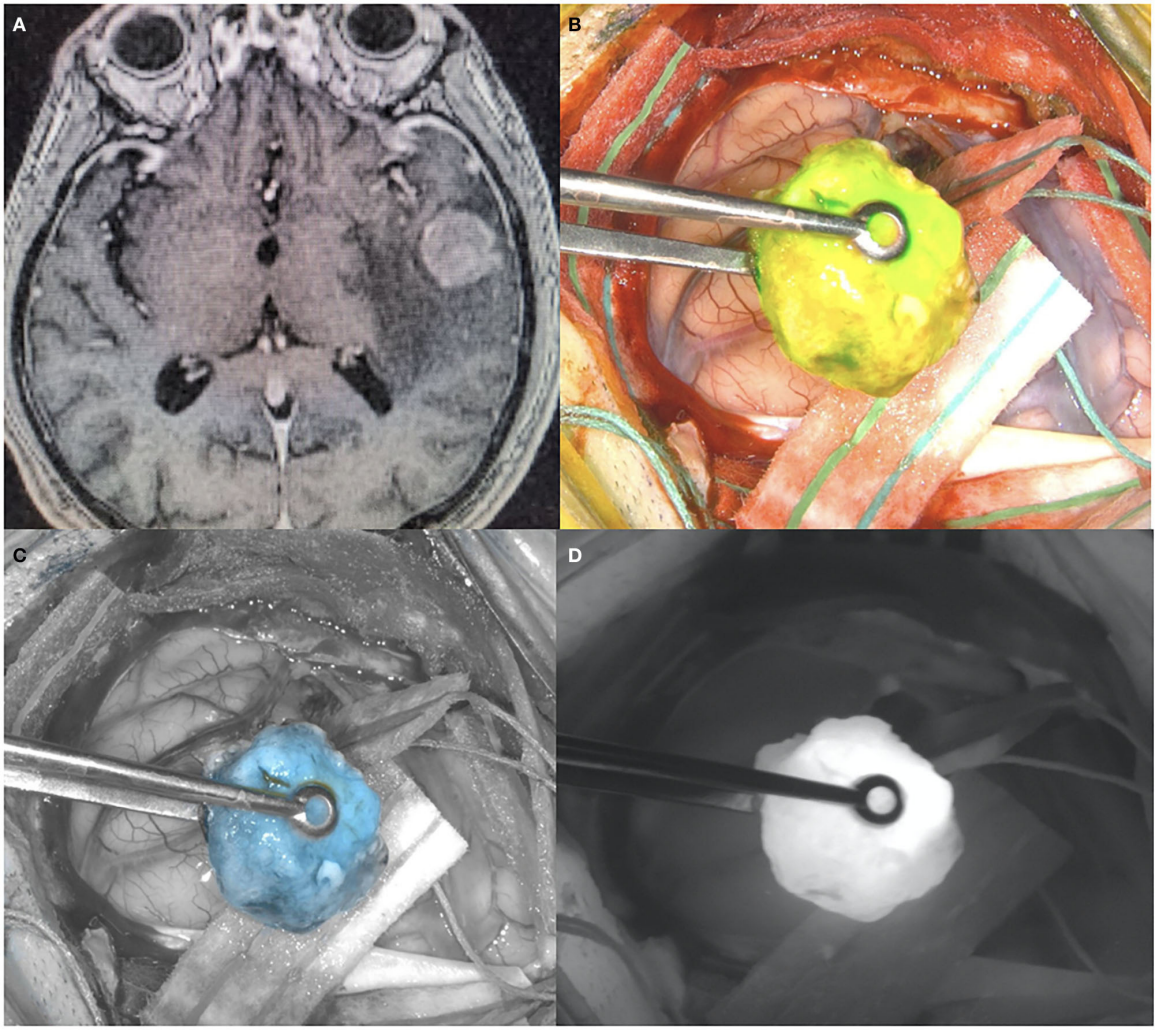
**(A)** Pre-operative T1 post-contrast MR, showing a left temporal lesion with surrounding vasogenic edema. Histological diagnosis showed a metastatic lung adenocarcinoma. **(B)** Intraoperative picture during the surgical removal of the same case depicted in **(A)**, showing the ICG uptake by the tumor visualized with yellow-green excitation. **(C)** Intraoperative picture during the surgical removal of the same case depicted in **(A)**, showing the ICG uptake by the tumor with blue excitation. **(D)** Intraoperative picture during the surgical removal of the same case depicted in **(A)**, showing the ICG uptake by the tumor visualized with near-infrared excitation.

Indocyanine Green (ICG) has been used widely in medical applications, beginning with hepatic and cardiac function measurement, and expanding into ophthalmology indications where it is FDA approved (Table 2). Upon systemic administration, as an amphiphilic, tricarbocyanine iodide dye, ICG binds to plasma proteins yielding many advantages including its confinement to the vascular compartment (19). Additionally, it's light excitation and emission in the near-infrared (NIR), low toxicity, and rapid excretion leads to its popularity. ICG has been used as a cerebrovascular intraoperative contrast agent to confirm aneurysm occlusion during surgery. More recently, a more delayed administration of ICG has been described prior to glioma surgery (20). ICG works as a passive targeting agent and requires the breakdown of the BBB to concentrate at the tumor site (19). Unlike 5-ALA and fluorescein, two popular FDA-approved fluorophore agents, ICG is a near-infrared (NIR) fluorophore which presents unique utility in labeling tumor tissue (15, 21). NIR imaging affords higher resolution with increasing tissue penetration depths with excellent signal to noise ratio (SNR) (Figure 1).

**Table 2:** Fluorophores currently used in brain tumor surgery and Food and Drug Administration (FDA) use approval.

**Agent**	**Year of FDA approval**	**Use of FDA approval**
ICG	1959	1.Determining cardiac output, hepatic function and liver blood flow. 2. For ophthalmic angiography.
Fluorescein	2006	1. Diagnostic fluorescein angiography or angioscopy of the retina and iris vasculature.
5-ALA	2017	1. Intraoperative optical imaging agent in patients with suspected high-grade gliomas

*FDA, Food and Drug Administration; ICG, indocyanine green; 5-ALA, 5-aminolevulinic acid*.

Both preclinical and clinical studies report the use of ICG for glioma surgery albeit within a few minutes after systemic administration exploiting vascular permeability of glioma compared to normal brain (19, 22). Haglund et al. used ICG for enhanced optical imaging in human gliomas (23). Hansen et al. found that when ICG was injected intravenously into tumor-bearing rats, the tumor fluoresced intensely at 60–120 mg/kg for at least 1 h after injection (24). This study showed the ability of ICG to distinguish rat brain tumor from normal parenchyma with adequate tumor to normal brain background ratio, with minimal post-resection residual tumor cells (24).

ICG intrinsic properties include a plasma half-life of 3–4 min; in preclinical studies after 10 min only a small amount of originally injected ICG volume can be detected in the blood (25). Due to this short half-life, ICG is usually given as a bolus dose of <0.5–1 mg/kg and NIR imaging is performed shortly after (23). Haglund et al. found that ICG fluorescence of glioma tissue, unlike fluorescein, is time-dependent (23). Martirosyan et al. found tissue fluorescence when ICG was injected 15 min prior to visualization, while Haglund et al. found an optical signal between 5 and 10 min after ICG injection (23, 25). The dye uptake rate was shown to be faster in HGGs compared to low-grade gliomas, and this diversity in tumor cell population may explain the time-dependent nature of the ICG peak signal. Studies suggest that as ICG is rapidly being eliminated from the blood at 18–24% per minute, the dye is sequestered into the tumor (23).

More recently, ICG has been administered at higher doses and tumor visualization has been improved by exploiting the enhanced permeability and retention (EPR) of nanoparticle-sized ICG fluorophore within brain tumors (26). Zeh et al. administered doses above the FDA-approved limit of 2 mg/kg in rodents implanted with human glioma xenografts and demonstrated a broad plateau period extending up to 72 h, thus allowing for optimal imaging of glioma as compared to normal brain (20). With the SWIG approach, combining the natural permeability of the tumor vascularity with poor clearance allows for high doses of ICG to penetrate the tumor, a high dose (5 mg/kg) of intravenous ICG is given a day (24 h) prior to surgery, yielding improved intraoperative visualization of the tumor compared to white light (15, 27, 28). In a series of 15 gliomas featuring 11 HGG, sensitivity was 98% and specificity 45% (27).

## Fluorescein Sodium

**Figure 2:**
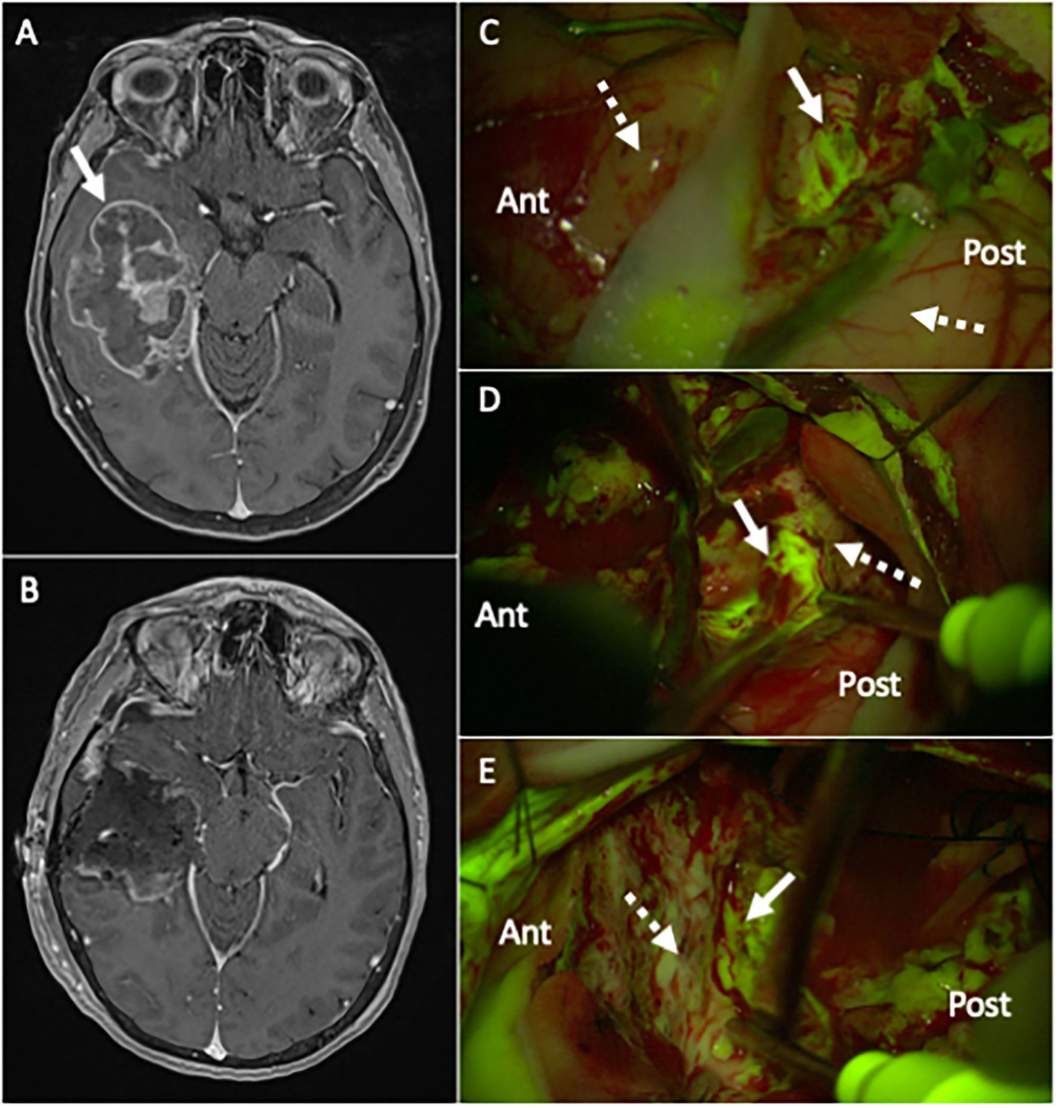
**(A)** Pre-operative T1 post-contrast MR, showing a large right temporal lesion (white arrow), with irregular enhancement and mass effect, compatible with the suspect of high-grade glioma. **(B)** Post-operative T1 post-contrast MR, performed 24 h after surgery, confirming a gross-total resection (GTR) of the lesion (histological diagnosis showed a Glioblastoma, IDH wild-type). **(C–E)** Intraoperative picture during the surgical removal of the same case depicted in **(A)**, taken with the Y560 filter activated (Pentero 900 microscope, Carl Zeiss Meditec, Oberkochen, Germany): after a small corticectomy **(C)**, the pathological tissue is clearly visible as a bright green-yellow fluorescent area (white arrow), while the non-pathological temporal cortex (dotted white arrow) anteriorly and posteriorly is non-fluorescent (for orientation, Ant is anterior and Post is posterior temporal lobe); during surgical removal with ultrasonic aspirator (Sonoca 300, Soring, Quickborn, Germany), subcortical tumoral tissue is clearly discernible from normal peri-tumoral parenchyma by its bright green-yellow fluorescence (white arrow in **(D)** at the posterior border and in **(E)** at the anterior border), compared to pinkish peritumoral parenchyma (dotted white arrow).

Fluorescein sodium (FS) is best known for its use in ophthalmology where it is FDA approved for angiography or angioscopy of the retina and iris vasculature (FDA approval letter). Recently, it has been reintroduced into neuro-oncologic surgery for HGG FGS. FS has a molecular weight of 376.27 and is the sodium salt of fluorescein. It was first used to visualize malignant brain tumor in 1948 (29). FS accumulates in HGGs where the BBB is disrupted and provides intra-operative visualization that is similar to pre-operative contrast-enhanced T1 images in which gadolinium accumulation is seen (30–33). FS can be viewed under white light, but the use of an operating microscope fitted with a dedicated filter allows to significantly reduce the dose needed to highlight tumoral tissue (Figure 2) (19, 30, 31, 34). FS is excited at 460–500 nm and emits a green, fluorescent emission wavelength at 540–690 nm (19, 35). FS is administered at the time of anesthesia prior to a craniotomy for HGG FGS. Immediately after systemic administration FS flows within the cerebral vasculature and afterwards accumulates in tumoral area where there is a damage of BBB. Fluorescence by FS can be visualized for up to 4 h after administration.

Both preclinical and clinical studies have confirmed that fluorescein, unlike other fluorophores, does not accumulate intracellularly but in the extracellular space in brain tumors (32, 35–37). Instead of tumor-specific uptake, it has been demonstrated that fluorescein sodium accumulates at disruptions of the BBB in areas with high- density tumor cells, thereby proving useful for HGG visualization (30–33, 36, 37). Fluorescein sodium has been found to have a sensitivity of 82–94% and a specificity of 90–91% for glioma visualization (19, 31, 32, 38). However, it has also been shown that FS fluorescence might not be limited to tumor tissue. As a matter of fact, some areas, such as dura mater, circumventricular organs and choroid plexus, due to the lack of BBB, appears intensively fluorescent (35). In addition, although the presence of FS fluorescence in normal brain parenchyma close to tumor tissue has been occasionally shown and considered a consequence of direct surgical manipulation (33, 39–41), it has been suggested that the application of a strict intraoperative protocol of FS injection could significantly limit this event (35).

Many studies have reported the safety, efficacy, and convenience of using fluorescein sodium during HGG resection (7, 16, 31, 35, 42–46). Maximal resection or gross-total resection (GTR) of tumors with FS has been reported with an increase in progression-free survival (PFS) (16, 19, 31–33, 35, 39, 40, 44, 45, 47). Additionally, due to concern that fluorescein is non-selective for tumor tissue, one study concluded that dual labeling with 5-ALA and fluorescein allowed for superior visualization in high-grade glioma resection because fluorescein sodium enhanced background tissue and 5-ALA enhanced tumor tissue (48).

Few studies have reported guidelines on when to administer FS for best visualization in the surgical field. Fluorescein distributes to tissues within 10 min and has a plasma half-life of 23.5 min (49). In clinical practice, FS is administered systemically in the operating room either immediately after anesthesia induction or prior to surgical resection (7, 16, 18, 31, 33, 35, 39, 42, 45, 49, 50). In order to elucidate whether fluorescein can be seen in non-tumor tissue, one study evaluated the pharmacokinetics of fluorescein sodium in different preclinical mouse models, without and with xenograft tumor (51). This study established that the majority (up to 70%) of injected fluorescein exists in circulating blood in the unbound form and that only up to 30% result to be protein-bound. Due to its low molecular weight, the circulating unbound form accumulates in normal mice brain tissue without xenograft tumor, with a peak timing of 30 min after injection, particularly if injected at human-equivalent high dosage, and that washout from normal brain seems to be completed 120 min after fluorescein injection. When used in orthotopic glioma models, the presence of unbound fluorescein in normal brain tissue 60 min after injection significantly reduces the tumor-to-normal contrast (51). The study also elucidated the fact that pegylated fluorescein sodium, which better matches gadolinium contrast in sizing, seems to provide more suitable kinetics and a higher ratio of tumor fluorescence compared to normal brain tissue in othotopic glioma models (51). There is a paucity of clinical data on the best timing of FS administration. Some studies have reported administration of fluorescein after incision of the dura with resection beginning as early as 10 or 20 min after injection (7, 16, 33, 39, 42, 44). However, in more recent experiences it has been suggested to use low dose (5 mg/kg) of fluorescein, with intravenous injection performed after patient intubation, thus in most of the cases around 1 h before incision of dura mater (18, 31, 35). More specifically, it has been shown that the optimal strategy to optimize fluorescent contrast during surgery is to use lower dose (1–5 mg/kg) to minimize unspecific extravasation, administered 2–4 h before visualization, which corresponds to the wash-out period of the FLS (43). Surely, a need for further investigation of the clinically-relevant pharmacokinetics of optimal fluorescein sodium administration remains.

## 5-Aminolevulinic ACID (5-ALA)

**Figure 3:**
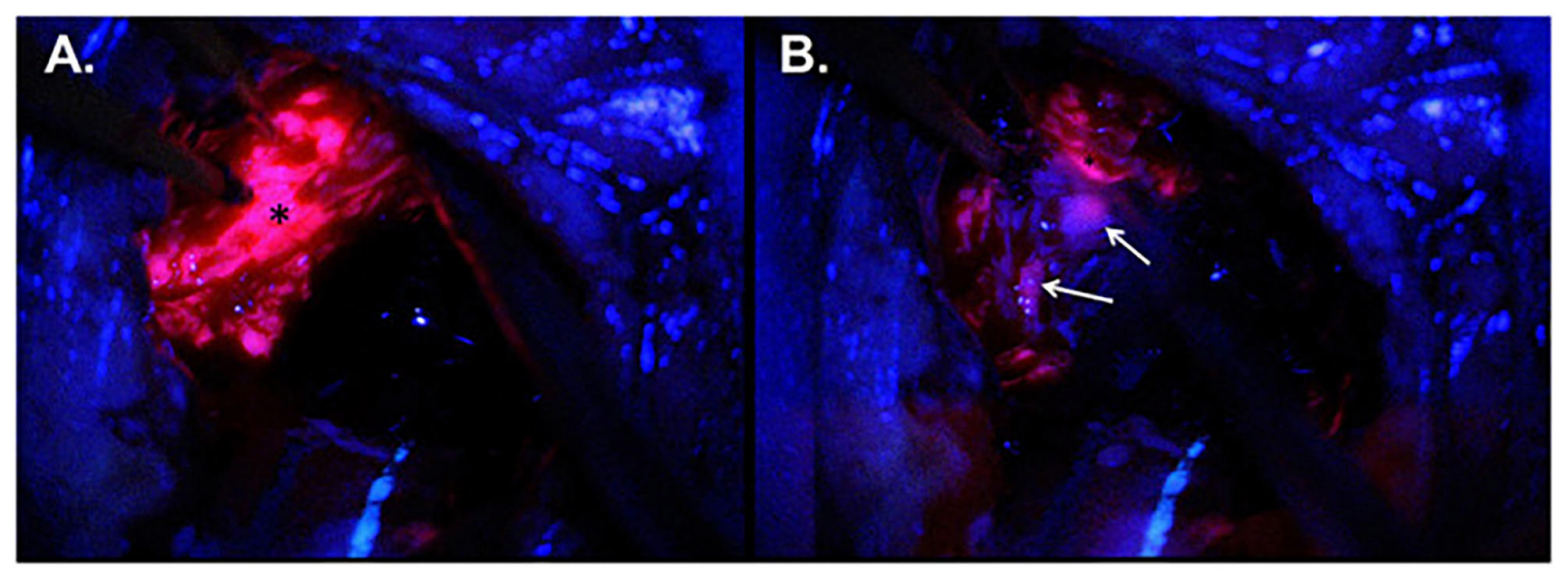
Intraoperative imaging for case demonstration patient. **(A)** Tumor bulk fluorescence after 5-ALA administration (asterisk). **(B)** Infiltrative margin fluorescence after 5-ALA administration (white arrows). Taken with permission from Maragkos et al. (53).

5-ALA, a precursor metabolized in the heme biosynthesis pathway to protoporphyrin (PPIX), accumulates intracellularly in tumor cells, and has a high affinity for high-grade glioma tissue (52). PPIX absorbs light between 375 and 440 nm and emits a violet-red fluorescence at 635 nm (Figure 3) (11) 5-ALA is the most well-studied fluorescent agent in glioma surgery that has been granted FDA approval (54, 55). A landmark randomized controlled study (RCT) was performed where HGG patients were randomized to FGS or conventional microsurgery (8) 5-ALA FGS was associated with improved progression-free survival (PFS) and greater overall tumor resection compared to white light control. 5-ALA FGS has been shown to be both safe and effective, with minimal side effects (8, 56, 57).

Since the publication of the RCT, the widespread use of 5-ALA globally has been based upon the same drug administration criteria used in the trial. An oral dose of ALA HCL solution of 20 mg/kg body weight, is administered 3 h (range 2–4 h) prior to induction of anesthesia (8) 5-ALA is rapidly absorbed into the blood after ingestion within 1 h and is metabolized quickly thereafter in brain tumors to its fluorescent PPIX metabolite. This dosing regimen was based upon rodent experiments in which there was a fluorescence peak observed 6 h after administration (10). The decision to have the patient ingest the medication at 3 h (with a range of 2–4 h) prior to surgery was recommended to allow ample time for anesthesia, monitoring and performing the craniotomy, in order for peak intraoperative PPIX fluorescence to be present during tumor resection (8, 56). As a result of this RCT and other European multicenter trials, the same administration criterion was used for the Food and Drug Administration (FDA) approval of 5-ALA (Gleolan®) in 2017 [(54); FDA approval] (58).

Recent studies have suggested that 5-ALA fluorescence may have a longer window of detection than previously described. In a prospective study of 68 patients and 201 tumor samples, Kaneko et al. found that maximal fluorescence intensity was observed 7–8 h following 5-ALA administration, and weak fluorescence peaked later than strong fluorescence, at 8–9 h (59). While prior animal studies have suggested an earlier fluorescence peak, there is now evidence that a longer latency time might lead to stronger fluorescence of HGG tissue. In a retrospective study of 16 patients who received 5-ALA over 4 h prior to anesthesia induction, our group found that adequate intraoperative fluorescence was seen up to almost 28 h post-ingestion, with no 5-ALA-related toxicity (53). Understanding the time window for 5-ALA PpIX fluorescence is clinically relevant, as surgeries are not uncommonly delayed due to emergent cases, staffing issues or other logistical challenges, and it is important to know if intraoperative fluorescence may be utilized despite delays in surgery. Unlike fluorescein and ICG which may be given in the operating room after induction of anesthesia, 5-ALA requires oral administration, and therefore there exists an element of anticipating time of surgery. Having a less narrow window of adequate intraoperative fluorescence allows surgeons greater flexibility with using 5-ALA for glioma surgery.

## Targeted Fluorophores

In addition to the fluorophores mentioned above, there are current studies on fluorophores with more directed mechanisms of action, such as specific receptor targets. Below is a description of several targeted agents currently under clinical investigation.

### BLZ-100

A conjugate of tumor-specific peptide chlorotoxin paired with a near-infrared fluorophore, BLZ-100 (tozuleristide, Blaze Bioscience Inc, Seattle, Washington) is visualized with a NIR camera (60). A recent phase I trial demonstrated safety and efficacy for the use of BLZ-100 in patients with primary and recurrent glioblastoma (60). Ongoing evaluation of the conjugate is currently being studied in both adult and pediatric brain tumors (NCT02234297, NCT02462629). BLZ-100 has the same benefits as ICG in the NIR spectrum, however, it adds further tumor specificity with chlorotoxin, a scorpion venom. Given intravenously, BLZ-100, also known as The Tumor Paint©, can safely be given to adults in doses ranging from 3 to 30 mg (60). In this trial, BLZ-100 was administered as a slow IV bolus injection over 1–5 min 3–29 h before surgery. It has a serum half-life of ~30 min, however, unlike 5-ALA, has been shown to be retained in tumors for over 24 h, particularly in doses over 9 mg or greater in World Health Organization (WHO) grade III and IV gliomas (60). Its length of time in tumor tissue is currently unknown. Further study is required on a larger patient sample, however, depending upon the sensitivity of specificity of BLZ-100, it may provide a useful fluorophore given its high intensity of fluorescence and prolonged retention in glioma tissue.

### Alkyl Phosphocholine Analogs

Alkylphosphocholine analogs (APCs) are small synthetic phospholipid ether molecules, which may target specific tumors types, including osteosarcoma, pancreatic adenocarcinoma and glioblastoma (61). Due to lipid raft expression, APCs are able to remain intracellular for prolonged periods of time (61), providing a theoretical advantage for FGS. To date, there are no published clinical results on APC use in gliomas, however, in a proof-of-principle preclinical study, high glioblastoma cell selectivity has been shown with two APCs (CLR1501 and CLR1502, Cellectar Biosciences, Madison, Wisconsin) (62). In the xenograft model, the two APCs were given intravenously in doses ranging from 2 to 16 mg/kg 4 days prior to sacrifice. CLR1501 (green spectrum) showed a tumor to brain fluorescence ratio comparable to 5-ALA, while CLR1502 (NIR) had a superior tumor to brain fluorescence ratio (62).

### Epidermal Growth Factor Receptor (EGFR) Targeted Fluorophores

Expressed as cell-surface receptors in many cancers including gliomas, epidermal growth factor receptor (EGFR) may be conjugated with fluorescent dyes, allowing for targeted cell-surface fluorescence (63). In an *in vivo* animal study of IRDye800CW labeled with anti-EGFR antibodies, there was a 100% sensitivity and specificity for distinguishing GBM-specific mutated EGFR positive from EGFR negative cell lines (64). While no clinical studies have been performed to date, preclinical studies have administered intravenous anti-EGFR fluorescence conjugates into rats, and observed safe doses up to 24.5 mg/kg of an IRDye800CW anti-EGFR antibody (ABY-029, Affibody, Sweden) (65). However, due to its short half-life it will need to be given during surgery in humans, and in preclinical investigation had poor fluorescent intensity in the brain (65). Based upon this preclinical trial, it was determined that ABY-029 can be administered within minutes to hours of the surgery start time, and fluorescence was still observed 48 h post-administration (66).

Cetuximab, an EGFR monoclonal antibody, when conjugated with fluorescent IRDye 800CW, fluoresces in the NIR, and has been shown in preclinical studies to be effective for glioma surgery (67). In a first-in-human study, cetuximab-IRDye800 demonstrated both safety and efficacy, with high signal to background ration aiding in glioblastoma visualization (68). Larger studies are warranted to better underwent the use of cetuximab conjugates for FGS.

## Choosing the Right Fluorophores: Proposed Suggestions

While all fluorophores provide varying degrees of intraoperative fluorescence during brain tumor surgery, the various mechanisms of action make certain fluorophores better choices for different tumor types. Below is an outline for the different fluorophores currently available for use in FGS, based upon tumor pathology.

### High-Grade Gliomas

High-grade gliomas (HGG) are the most widely studied tumor type in FGS. The three most commonly studied fluorophores, 5-ALA, fluorescein and ICG, have all been used in HGG surgery. 5-ALA has been the most robustly studied fluorophore in malignant gliomas. PPIX specifically accumulates in the tumor intracellular space, resulting in a robust red fluorescence in the tumor bulk, and a surrounding lighter pink fluorescence peripherally, representing surrounding infiltrative tumor cells (69, 70). This selective uptake in tumor cells results in high sensitivity and specficity, with prior studies showing mean sensitivity and specificity for delineating tumor tissue vs. surrounding brain to be 83–87% and 89–100%, respectively (6, 10, 71, 72). Additionally, 5-ALA has a strong positive predictive value, with multiple studies finding 100% PPV for solid fluorescence in both primary and recurrent HGG (70, 73, 74). Beyond histologic accuracy of 5-ALA PPIX fluorescence, correlations have been made between fluorescence intensity and histological grading, suggesting the ability to approximate grade by fluorescent signal (75, 76).

In addition to being safe for use in HGG surgery, 5-ALA has also shown to improve patient outcomes. In a phase III randomized controlled trial of 322 HGG patients randomized to 5-ALA FGS or white light surgery, Stummer et al. found that patients who received 5-ALA experience more complete resections of contrast-enhancing tumor (65 vs. 36%, *p* < 0.0001), and higher 6-month progression-free survival (41.0 vs. 21.1%, *p* = 0.0003) (8). In a retrospective study of glioblastoma patients that had undergone surgery with 5-ALA, those patients without residual tissue fluorescence had greater median overall survival compared to those with residual fluorescence (27 vs. 17.5 months, *p* = 0.015) (77).

Despite the advantages of 5-ALA for HGG surgery, the agent is expensive and not universally available. Following a series of successful clinical trials in 5-ALA, FGS researchers tested the safety and efficacy of other more widely accessible fluorophores for HGG resections. Several recent studies have assessed the use of fluorescein sodium, and found that fluorescein improves extent of resection and rates of gross total resection, with GTR rates up to 80% (7, 30, 31, 78). Despite being selective for areas of blood-brain barrier breakdown, rather than tumor tissue itself, fluorescein fluorescent signal has been shown to correlate with contrast-enhancement on preoperative MR imaging (32, 40). To date, there are no randomized controlled trials for fluorescein-guided tumor surgery, however, in a recent multicenter phase II study, Acerbi et al. found that fluorescein was safe and effective in HGG surgery, with a GTR rate of 82.6% and 6-month progression-free survival of 56.6% (35). ICG, a fluorophore used primarily in angiography, has recently been implemented in brain tumor surgery, for its enhanced permeability retention (EPR) effect as a result of BBB disruption. The second window ICG (SWIG) technique allows for ICG accumulation in HGG tissue, allowing for improved visualization (15, 28). While this technique has not yet been widely studies, in a recent case series of 11 HGG, SWIG was found to be highly sensitive for HGG tissue, and fluorescent signal correlated with MR imaging (27). While safely tolerated, the major disadvtange of ICG is that unlike 5-ALA and fluorescein, it emits in the near infrared spectrum, making it logistically difficult to operate while visualizing the signal.

### Low-Grade Gliomas

Low-grade gliomas have created a larger challenge for FGS compared to HGG, as the lower grade tumors do not have as robust areas of BBB breakdown and contrast uptake compared to more infiltrative lesions. While historically “watch and wait” was the treatment paradigm for these lesions, there is now strong evidence for early, maximal safe resection (74, 79, 80). However, these tumors may be difficult to remove completely, as their appearance may only appear slightly different compared to normal brain, and therefore 5-ALA has been implemented. While initially researchers were doubtful that there would be any 5-ALA PPIX uptake in LGG cells due to less BBB breakdown, the differences appear quantitative, rather than qualitative (81). Although initial studies showed no fluorescence in LGG patients (82, 83). Widhalm et al. found a subset of non-contrast-enhancing LGG patients with 5-ALA-induced fluorescence (84). In the largest series to date, Jaber et al. found visible fluorescence in only 13 of 82 WHO grade II gliomas, and concluded that the majority of LGG do not show visible fluorescence (85). However, the value in use of 5-ALA for LGG surgery may be in the histological heterogeneity of these tumors, as areas of malignant transformation, or anaplastic foci, are characteristic for LGGs (86). By fluorescing areas of contrast-enhancement, 5-ALA can help identify areas of higher metabolic activity and subsequently greater proliferation (87).

### Meningiomas

Meningiomas are the most common benign brain tumors, and with the propensity to enhance on contrasted imaging, they have been studied in the field of FGS. While the three major fluorophores, 5-ALA, ICG, and fluorescein, have all been studied in the visualization of meningiomas, 5-ALA has been most widely studied, with over 10 clinical studies to date (88). The largest study of FGS use in meningiomas is by Millesi et al., where after administration of 5-ALA to 204 meningioma patients, visible fluorescent signal was found in 91% of tumors, and 100% positive preductive value for bone infiltration (89). In a study of 12 meningioma patients, seven skull base and five convexity, Della Puppa et al. found 89% sensitivity and 100% specificity for 5-ALA detecting bone invasion (90). Additionally, in a small series of eight intradural spinal meningiomas using 5-ALA, there was a positive predictive value of 100% (91).

ICG and fluorescein have also been used in the resection of meningiomas. ICG in particular has been found to be helpful in cases where the tumor invades the dural sinuses, and helps visualize venous collaterals, the surrounding patent sinuses and the pial vascular supply (92–94). In these cases, however, ICG is being used as a vascular contrast agent with visualization within minutes of IV bolus dosing. In contrast, Lee et al., employed the high dose, delayed second window ICG technique found that 78% meningiomas exhibited more fluorescent signal than surrounding brain, with a sensitivity of 96.4%, but only 39% specificity (95). Fluorescein, like ICG, accumulates within tumor regions rapidly, however, the fluorescent signal may persist for hours, aiding in the visualization of meningiomas (54). In a recent study of 30 patients with meningiomas undergoing resection, Akcakaya et al. found that 88% of meningiomas demonstrate diffuse homogenous intraoperative enhancement, yielding a resection rate of 87% (96). With fluorescein, confocal microscopy can help illuminate meningiomas on the cellular level, with a confocal/histology concordance of 90%, with identification of dural invasion (97).

### Brain Metastases

As the most common brain tumors in adults, the treatment paradigm for cerebral metastases has been widely studied. Depending on the size, location and quantity of CNS metastatic lesions, treatment options may include chemotherapy, radiation modalities such as stereotactic radiosurgery (SRS) and whole brain radiation therapy (WBRT), and surgical resection. For symptomatic tumors causing mass effect and cerebral compression, complete resection of metastatic lesions is always the goal when attainable (98). Despite the non-infiltrative nature of these lesions (compared to gliomas), 30% of resections are incomplete, which may explain the 60% local recurrence rate (99, 100), providing a rationale for use of an adjunct such as FGS.

5-ALA has been shown to fluoresce in metastatic lung, breast, colon, bladder, melanoma and other primary cancers with brain metastases (101–104). First studied in 2007, 5-ALA for brain metastases has not been widely studied, with only several large patient studies (54, 105). In the largest experience to date of 157 patients with known metastatic lesions undergoing resection with 5-ALA, Marhold et al. concluded that 66% of tumors exhibited fluorescence, with ductal breast cancer having the highest rate of fluorescence (106). In addition to having variable rates of fluorescence, 5-ALA has shown fluorescence in extra-tumoral edema in cases where the tumor core demonstrated poor fluorescence, making FGS less reliable in these cases (107). Further study is warranted on determining predictors of fluorescence among different primary cancers with brain metastases, as well as a more specified use for FGS in metastatic disease.

Fluorescein sodium has been broadly studied for its use in resection of brain metastases. In a series of 30 patients, Schebesch et al. found fluorescence in 90% of tumors, leading to an 83.3% GTR rate (108, 109). In a follow up study expanding upon this initial experience, Hohne et al. found a 95% fluorescence rate in 95 patients, with an 86% rate of no residual contrast enhancement. In this series, lung adenocarcinoma, melanoma and renal cell carcinoma were the only primary cancers that did not consistency exhibit fluorescein fluorescence (110). As part of the FLUOCERTUM Prospective Study, 25 metastastic lesions were included, of which 24 showed heterogenous enhancement (111). From these primary experiences and others, fluorescein appears to be a reliable fluorophore for brain metastasis surgery.

Second Window ICG technique appears to have advantages for visualization of brain metastasis as most of these tumors have a disrupted BBB and thus accumulate ICG (Figure 1). In a recent publication, Teng et al. demonstrated improved survival in patients with resection of all near infrared fluorescent signal in patients undergoing craniotomy for brain metastasis (112). In 47 patients who underwent resection of 51 metastatic lesions, all tumors demonstrated quantifiable NIR fluorescence with a mean SBR of 4.9. Diagnostic accuracy was improved by changing the threshold signal as compared to normal brain background, and a lack of residual NIR fluorescence not only predicted the postoperative MRI gadolinium findings but also predicted progression free survival at 1 year. ICG may prove to have its greatest role in resection of these tumors.

### Pediatric Brain Tumors

Similar to the adult literature, maximal safe resection is the standard of care in pediatric HGG. However, unlike the adult literature, we lack randomized controlled trial data for using FGS in the treatment of pediatric brain tumors. The use of 5-ALA for brain tumor surgery in children was first reported by Ruge et al., where 5-ALA was used to resect in a 9-year-old with a right temporal lobe pleomorphic xantoastrocytoma (PXA) (113). There have been no large single center studies currently, however, in 78 patients among 20 European centers, Stummer et al. found that 85% HGG and 80% ependymomas showed fluorescence, and that most primitive neuro-ectodermal tumors (PNET), gangliogliomas, medulloblastomas and pilocytic astrocytomas did not fluoresce (70). Other studies have shown robust fluorescence in HGG, with inconsistent fluuoresence in medulloblastoma, which may be related to underlying subtype (21, 114). While the safety data for 5-ALA use has been well-established in the adult literature, it is less studied in pediatric patients, and there has prior suggestion of increased liver enzyme values with decreasing patient age (115). In a multicenter study of 24 pediatric patients, de Laurentis et al. found fluorescence in 77.8% of tumors, and found fluorescence to be “helpful” in half of the cases (116). BLZ-100 has recently shown to be safe and effective in the treatment of both HGG and LGG in adults, and is currently under investigation for pediatric brain tumors (60).

### Spinal Cord Intramedullary Tumors

In the resection of intramedullary spinal cord tumors, establishing the margin between tumor and spinal cord is essential to minimize the risk of spinal cord injury. Ependymomas and astrocytomas, two of the most common intramedullary tumors, both have robust uptake of 5-ALA, and therefore it has studied for this use. Gross total resection of ependymomas is especially crucial, as it is a potentially curable lesion. The first series of intramedullary ependymomas using 5-ALA was published in 2013, when Inoue et al. found that 77% of ependymomas demonstrated fluorescence, leading to a GTR in 80% of cases (117). In a study of 52 patients with 55 spinal tumors including 12 ependymomas, Millesi et al. found 5-ALA to be a safe and effective adjunct in cases of ependymomas, meningiomas, hemangiopericytomas and drop metastases, with a GTR rate of 75% ependymomas (118). With spinal astrocytomas, the margins are less defined, and surgeons are faced with a more difficult situation in where to end a subtotal resection, at the expense of sensorimotor function. 5-ALA may help in identifying tumor margins, and to aid in deciding where to limit cytoreduction, to preserve high-level functionality.

Fluorescein sodium has been shown to be effective for intramedullary tumors in several studies. Acerbi et al. found fluorescence in 82% of tumors, including ependymomas, hemangioblastomas, astrocytoma, and a glioneuronal tumor forming rosettes (119). In a study of 34 intradural extramedullary and 15 intramedullary lesions, surgeons found fluorescein helpful in delineating tumor from surrounding tissue in 96% cases (120).

ICG has additionally been used for intramedullary tumors, specifically vascular spinal cord lesions, such as hemangioblastoma and cavernous angioma (54). In these cases, identifying the feeding and draining veins of these tumors can help surgeons safely remove the lesion. In the case of cavernous angiomas, the surrounding spinal cord takes up the ICG fluorescence, however, the angioma remains avascular, providing surgeons with a nice margin for resection (121).

### Primary CNS Lymphoma and Stereotactic Biopsy

Safety and accuracy are the two most important objectives for stereotactic biopsy of cerebral lesions. Fluorescein has been shown to be effective in predicting tumor pathology, especially in contrast-enhancing lesions (122), and in a proof-of-concept study, Lynagh et al. found that *in vivo* fluorescein signal was strongly predictive of tumor tissue, and could be used to identify tumor prior to biopsy (123). Additionally, 5-ALA and ICG have been shown to be effective in identifying tumoral vessels, and 5-ALA, ICG and fluorescein can accurately detect tumor tissue prior to biopsy (124). By coupling fluorescence with confocal microscopy, identifying tumor tissue may now be feasible in real time without a frozen section (124), and may even increase the diagnostic yield, with Malinova et al. finding that 5-ALA fluorescence had a higher sensitivity and negative predictive value for unclear cerebral pathologies compared to frozen section (125). Second Window ICG has also been successfully used in stereotactic biopsy procedures, to assure the surgeon that the contrast-enhancing portion of the tumor has been biopsied (126).

Several case series have shown a potential role for 5-ALA PpIX fluorescence in primary CNS lympoma (127, 128). Lymphoma may mimic HGG radiographically, and therefore PpIX and other fluorophores have been used in cases of presumed HGG. However, the standard of care for primary CNS lymphoma is chemotherapy and radiotherapy following diagnostic biopsy (129), creating a potential role for 5-ALA in improving the diagnostic yield of lymphoma tissue. In the largest series to date on stereotactic biopsies for primary CNS lymphoma, Yamamoto et al. found that 34 of 41 lesions showed fluorescent signal, with a true-positive rate of 82.9% (128). Kiesel et al. found fluorescence in 79% primary lymphoma lesions, and it has recently been proposed that if a lesion demonstrates strong fluorescence, that it may not require intraoperative histopathology, potentially making biopsies safer by decreasing number of biopsy samples and reducing length of stay (130). Fluorescein has also been used to effectively delinate lymphoma tissue vs. normal brain in two case series, where lesions were preoperatively diagnosed as HGG (47, 108). Further study is required to better understand the role for fluorescein in primary CNS lymphoma. In addition, Second Window ICG accumulates dramatically as expected in primary CNS lymphoma, and may be effective in identifying this tissue for biopsy (131).

### Other Tumors and Pathological Conditions Requiring Surgery

In addition to the aforementioned CNS tumors, there are several other tumor types that have been studied in the FGS literature.

Peripheral nerve sheath tumors (PNST), comprised of mostly schwannomas, may be difficult to distinguish from surrounding tissues, allowing for a potential role for FGS guidance. In a series of 25 PNST, Vetrano et al. found that fluorescein showed fluorescence in 13 of 14 schwannomas, and allowed for further resection leading to a GTR in six neurofibroma cases and one schwannoma case (132). In a series by Marbacher et al. of 458 tumors receiving 5-ALA PpIX, zero of seven schwannomas showed fluorescence (133). To our knowledge, there have not been studied conducted evaluating SWIG for these tumors.

Sellar tumors, in particular pituitary adenomas, have not been reported to demonstrate strong fluorescent signal, and therefore, not much research has been studied on these tumors. In the Marbacher et al. series, only one of 12 pituitary adenomas demonstrated fluorescence (133). Falco et al. found fluorescence in one of one patients studies in the FLUOCERTUM cohort (111). The role of SWIG in these lesions may be limited, as the pituitary gland is a normal structure that fluoresces with ICG (54). Despite this potential limitaions, ICG endoscopy has been proposed for resection of skull base surgery, and techniques for use have been described (134), with distinguishable margins able to be identified in both functional and non-functional pituitary lesions (135).

Hemangioblastomas may be an important tumor for FGS, as subtotal resection may lead to local recurrence, and FGS may help visualize the intramural nodule within the associated peritumoral cyst. As part of the FLUOCERTUM prospective study of 279 patients receiving fluorescein-guided tumor resection, seven patients with hemangioblastoma were included, all of which demonstrated fluorescence (111). Utsuki et al. used 5-ALA fluorescence, and showed strong fluorescence in all nine hemangioblastoma cases (136). The use of ICG has been rarely reported for both brain and intramedullary hemangioblastoma resections (137, 138).

Several other tumor types have shown potential promise in FGS, such as strong fluorescence observed in fourth ventricle subependymomas (139), germ cell tumors undergoing endoscopic biopsy (140). Further research is warranted on these rarer tumors, to better understand which fluorophores may be implored for use.

## Conclusion

Understanding the administration and properties of the various fluorophores used in glioma surgery is essential for its use as a surgical adjunct. Fluorophores may target areas of blood-brain barrier breakdown, areas of inflammation, or specifically glioma cells. Fluorophores are administered by different routes, and surgeons should be aware of the relationship between fluorophore administration and tumor fluorescence. ICG and fluorescein work by passive targeting, and have the benefit of IV administration in the operating room within seconds (ICG as vascular angiography agent) to a couple hours (fluorescein), to the next day (Second Window ICG as an EPR accumulated agent). As an oral agent, 5-ALA is consumed prior to anesthseia, and is able to persist in target tissue for longer, allowing for logistical delays. Newly studied molecular targets, such as BLZ-100 and EGFR conjugates, are also given in the operating room, but may last in tumor tissue for up to several days.

## Author Contributions

AS and CH: conceptualization and investigation. AS, MR, NM, RB, JL, FA, and CH: writing—original draft preparation, writing—review, and editing. AS: visualization. CH: supervision and project. All authors have read and agreed to the published version of the manuscript.

## Conflict of Interest

CH is a consultant for NX Development Corporation (NXDC) and Synaptive Medical. NXDC, a privately held company, markets Gleolan (5-ALA, aminolevulinic acid hydrochloride). Gleolan is an optical imaging agent approved for the visualization of malignant tissue during glioma surgery. CH is a consultant for NXDC and receives royalty payments for the sale of Gleolan, has also received speaker fees by Carl Zeiss and Leica. FA has received speaker's fees from Carl Zeiss Meditec, Oberkochen, Germany. The remaining authors declare that the research was conducted in the absence of any commercial or financial relationships that could be construed as a potential conflict of interest.
